# Characterization of cancer-associated fibroblasts (CAFs) and development of a CAF-based risk model for triple-negative breast cancer

**DOI:** 10.1186/s12935-023-03152-w

**Published:** 2023-11-25

**Authors:** Ganggang Wang, Hao Zhang, Xiaowei Shen, Wenzhi Jin, Xiaoliang Wang, Zhijie Zhou

**Affiliations:** 1 Department of Hepatobiliary Surgery, Shanghai Pudong Hospital, Fudan University Pudong Medical Center, Shanghai, 201399 China; 2 Department of General Surgery, Qingpu Branch of Zhongshan Hospital, Fudan University, Shanghai, China

**Keywords:** Triple-negative breast cancer, Tumor microenvironment, Cancer-associated fibroblast, Prognosis, GPR34

## Abstract

Triple-negative breast Cancer (TNBC) is a highly malignant cancer with unclear pathogenesis. Within the tumor microenvironment (TME), cancer-associated fibroblasts (CAFs) vitally influence tumor onset and progression. Thus, this research aimed to identify distinct subgroups of CAF using single-cell and TNBC-related information from the GEO and TCGA databases, respectively. The primary aim was to establish a novel predictive model based on the CAF features and their clinical relevance. Moreover, the CAFs were analyzed for their immune characteristics, response to immunotherapy, and sensitivity to different drugs. The developed predictive model demonstrated significant effectiveness in determining the prognosis of patients with TNBC, TME, and the immune landscape of the tumor. Of note, the expression of GPR34 was significantly higher in TNBC tissues compared to that in other breast cancer (non-TNBC) tissues, indicating that GPR34 plays a crucial role in the onset and progression of TNBC. In summary, this research has yielded a novel predictive model for TNBC that holds promise for the accurate prediction of prognosis and response to immunotherapy in patients with TNBC.

**Supplementary Information:**

The online version contains supplementary material available at 10.1186/s12935-023-03152-w.

## Introduction

Triple-negative breast cancer (TNBC) is a fatal malignancy. It poses a significant challenge to treatment owing to the lack of definite targets and poor tumor microenvironment (TME) [1]. In this disease, estrogen receptor (ER), progesterone receptor (PR), and human epidermal growth factor receptor 2 (HER2) are negatively expressed. TNBC exhibits high histological grade, positive lymph node metastasis rate, and propensity for recurrence and distant metastasis.

The TME, comprising cancer, stromal, infiltrating immune, and other supportive cells, vitally regulates the onset and progression of tumors [2]. Among various solid tumors, cancer-associated fibroblasts (CAFs) are the most prevalent stromal cells [3]. CAFs significantly promote multiple pro-tumorigenic processes, including extracellular matrix remodeling, angiogenesis, cancer cell proliferation, inflammation, infiltration, metabolic reprogramming, resistance to chemotherapy, and evasion of immune cells [4, 5]. CAFs interact with malignant cells in breast cancer to coordinate breast cancer metastasis [6–8]. Additionally, CAFs have been involved in regulating immune suppression and chemoresistance, rendering them an innovative and promising target for anticancer therapy in advanced-stage breast cancer [9–13].

**Fig. 1:**
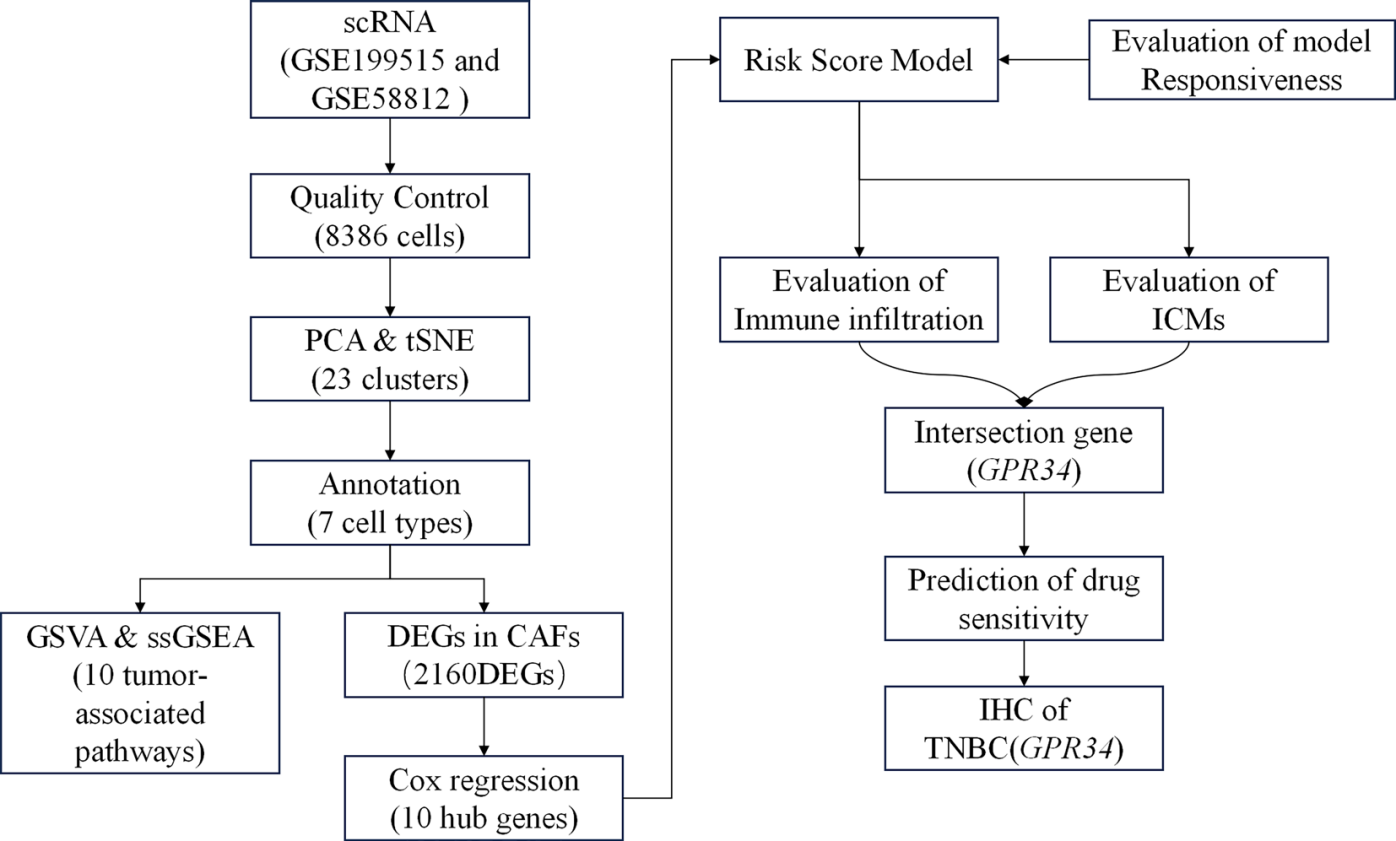
The study flow chart

Despite numerous studies on CAFs in TNBC, the systematic characteristics of CAF and their association with TNBC prognosis and response to immunotherapy response require further understanding. In this study, several databases were searched to retrieve TNBC single-cell transcriptome and RNA sequencing (scRNA-seq) data. Then, the CAF subsets and their association with TNBC risk features were identified. Furthermore, the CAF-based signatures were assessed for their clinical significance. This was followed by investigating the CAFs for their immune characteristics, response to immunotherapy, and sensitivity to drugs. A novel predictive model was established by integrating CAF-based risk attributes and clinical pathological traits, which aids in the clinical application of CAF features for TNBC prognosis. Insights into TNBC pathophysiology could be potentially gained through this constructed model, enabling highly targeted treatment, and improving the prognosis of patients with TNBC. The enriched key molecules were also validated to identify new therapeutic targets for targeted therapy in TNBC. The flow chart of this study was shown in Fig. 1.

## Materials and methods

### Data collection and processing

The scRNA-seq data were retrieved from the GSE199515 and GSE58812 datasets of the Gene Expression Omnibus (GEO) database (Additional file 4: Table S1). Data preprocessing steps involved the initial screening of single cells, requiring each gene to be expressed in a minimum of three cells and each cell to express in a minimum of 250 genes. The PercentageFeatureSet function in the R Seurat was employed to determine the rRNA and mitochondrial proportions. Single cells were subsequently analyzed, applying a minimum expression threshold for 6000 genes with UMI exceeding 100. This process yielded a total of 8386 cells for subsequent analyses.

### Definition of CAF

To comprehensively characterize the CAF signature in TNBC, the scRNA-seq data were re-assessed by employing the Seurat package [14]. Cells that expressed either more than 6000 or less than 250 genes were initially excluded, and the remaining genes were subjected to log normalization. The uniform manifold approximation and projection approach was used for non-linear dimensional reduction, with 15 principal components and a resolution of 0.2. Subsequently, distinct subgroups were established by clustering single cells through the FindNeighbors and FindClusters functions (dim = 40 and resolution = 0.2). The dimensional reduction was accomplished by the t-distributed stochastic neighbor embedding (t-SNE) using the RunTSNE function. Four marker genes, namely *ACTA2*, *FAP*, *PDGFRB*, and *NOTCH3*, were used to annotate the fibroblasts. Following that, re-clustering of the fibroblasts was completed using the identical FindNeighbors and FindClusters algorithm, followed by a t-SNE dimensionality reduction of the fibroblast clusters.

The marker genes for each CAF cluster were identified by utilizing the FindAllMarkers function, where a single cluster was compared with others using logFC = 0.5, minpct = 0.35, and a modified *P*-value of less than 0.05. Moreover, the Kyoto Encyclopedia of Genes and Genomes (KEGG) enrichment analysis was conducted on the marker genes of CAF clusters using the clusterProfiler package [15]. Lastly, the CNV features of the CAF clusters were examined using the CopyKAT R package to distinguish cancerous cells from healthy cells in each sample [16].

### Detection of hub genes in CAFs

The limma package aided in the identification of differentially expressed genes (DEGs) between healthy and cancerous tissues with the criteria of a false discovery rate threshold of < 0.05 and |log2(Fold Change)| > 1 [17]. Subsequently, the association of these DEGs with CAF clusters was examined, leading to the identification of key CAF-linked genes with *P* < 0.001 and cor > 0.4. The identification of genes associated with prognosis was carried out by means of univariate Cox regression analysis with the survival package, with a significance level of *P* < 0.05 (https://rdocumentation.org/packages/survival/versions/2.42-3). The least absolute shrinkage and selection operator (Lasso) Cox regression analysis was conducted to minimize the number of genes. Following this, a multivariate Cox regression analysis was conducted using a stepwise regression approach. According to the findings from the multivariate Cox model, a risk signature was developed using the following formula: risk score = Σβi * Expi, where ‘i’ denotes the gene in the risk signature, Expi denotes the levels of gene ‘i’, and βi denotes the coefficients of gene ‘i’ in the multivariate Cox model. After applying zero-mean normalization, patients were classified into high- and low-risk groups. The predictive capacity of the risk signature was assessed through receiver operating characteristic curve (ROC) analysis with the aid of the timeROC package (https://cran.r-project.org/web/packages/timeROC/index.html). This analysis process was similarly executed in the validation cohort.

### Immune landscape analysis

The CIBERSORT algorithm was employed to assess the proportions of 22 immune cell subtypes in the TCGA cohort. This method is instrumental in evaluating immune cell infiltration. To further examine the TME, the ESTIMATE algorithm (https://sourceforge.net/projects/estimateproject/) was utilized to calculate immune and stromal scores.

### Response to immune checkpoint blocks

The IMvigor210 cohort was utilized to retrieve transcriptomic data. Moreover, the GSE78220 cohort, including transcriptomic information from pre-treatment melanomas undergoing anti-PD-1 checkpoint inhibition treatment [18], was further assessed. The data were retrieved to ascertain the potential significance of the risk signature in predicting the response to immune checkpoint blocks.

### Clinical specimens

Specimens from 18 TNBC patients and 8 non-TNBC patients admitted for surgical intervention at the hospital were collected. Informed written consent was provided by all study subjects, and the study protocol was approved by the Ethics Committee of the hospital. Immunohistochemistry (IHC) using human microarrays was conducted to measure GPR34 protein expression.

### IHC

The TNBC and normal adjacent tissues were embedded in paraffin and sectioned. An anti-GPR34 antibody (#DF4972; Affinity) was used for staining, followed by exposure to an anti-IgG HRP-conjugated antibody (Biotime, China). The H-score system was utilized to evaluate the proportion of cells that stained positive and to investigate immunoreactivity. The expression analysis utilized the AI-based digital pathology image analysis software Aipathwell, developed by Servicebio. This software was used to calculate the H-score, which is a system employed to assess the proportion of positively stained cells and examine immunoreactivity. The H-score is determined using the following formula: H-SCORE = ∑(pi×i) = (percentage of weak intensity × 1) + (percentage of moderate intensity × 2) + (percentage of strong intensity × 3) [19].

### Statistical analysis

Statistical analyses were conducted employing R software (v3.6.3). Correlation matrices were developed using Pearson’s or Spearman’s correlation methods. For comparative analysis between the two groups, the Wilcoxon technique was implemented. Survival differences were compared using Kaplan–Meier curves with a log-rank test. The data with a *P*-value < 0.05 held statistical significance.

## Results

### Screening CAFs in scRNA-seq samples

**Fig. 2:**
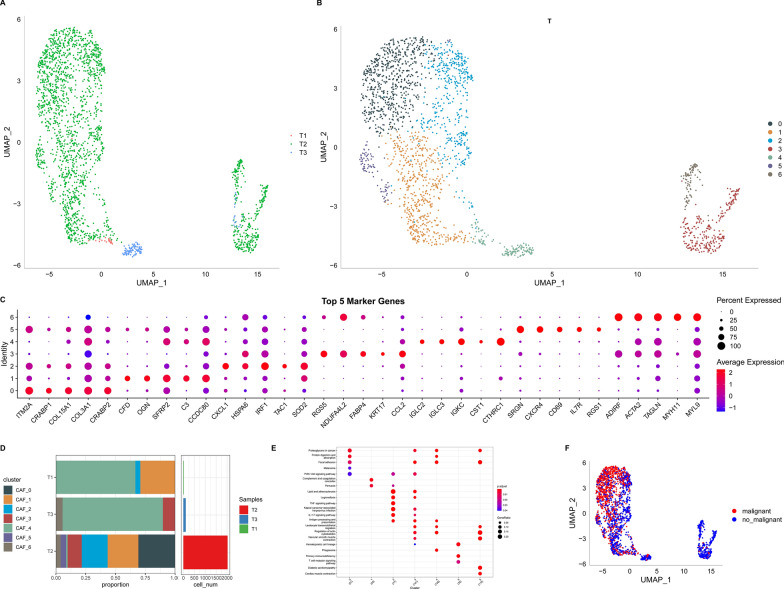
Detection of CAF cell clusters using the scRNA database of TNBC-affected individuals. **A** t-SNE plot showing the distribution of three samples; **B** distribution of seven CAF clusters; **C** expression profiles of the five leading marker genes in each of the seven clusters; **D** percentage and cell count of subgroups both in cancerous and nearby tissues; **E** KEGG enrichment analysis of seven fibroblast subgroups; **F** t-SNE plot displaying the malignant and non-malignant cell distribution in clustered cells

The scRNA-seq data yielded 8386 cells after the first screening stage. Following log-normalization and dimensionality reduction, 23 subpopulations were screened. Based on four marker genes, *ACTA2, FAP, PDGFRB*, and *NOTCH3*, seven CAF populations (Additional file 1: Fig. S1A, B) were identified. Furthermore, clustering and dimensionality reduction were performed on the cells from these seven CAF populations, yielding seven CAF clusters (Additional file 1: Fig. S1C, D). Figure 2A presents the t-SNE plot of the sample distributions, validating the presence of seven distinct clusters (Fig. 2B). In total, 1155 DEGs across the seven CAF clusters were found. Figure 2C presents the expression profiles of the top five DEGs, deemed as marker genes for these clusters.

Figure 2D presents the proportion of the seven clusters within each cohort. Moreover, KEGG analysis outcomes highlighted the enrichment of the DEGs in various pathways such as focal adhesion, vascular smooth muscle contraction, oxytocin, and PPARG signaling pathway (Fig. 2E). Additionally, based on CNV characteristics, the seven CAF clusters comprised 2155 cancerous and healthy cells (Fig. 2F).

### Expression of cancer-associated pathways in CAF

**Fig. 3:**
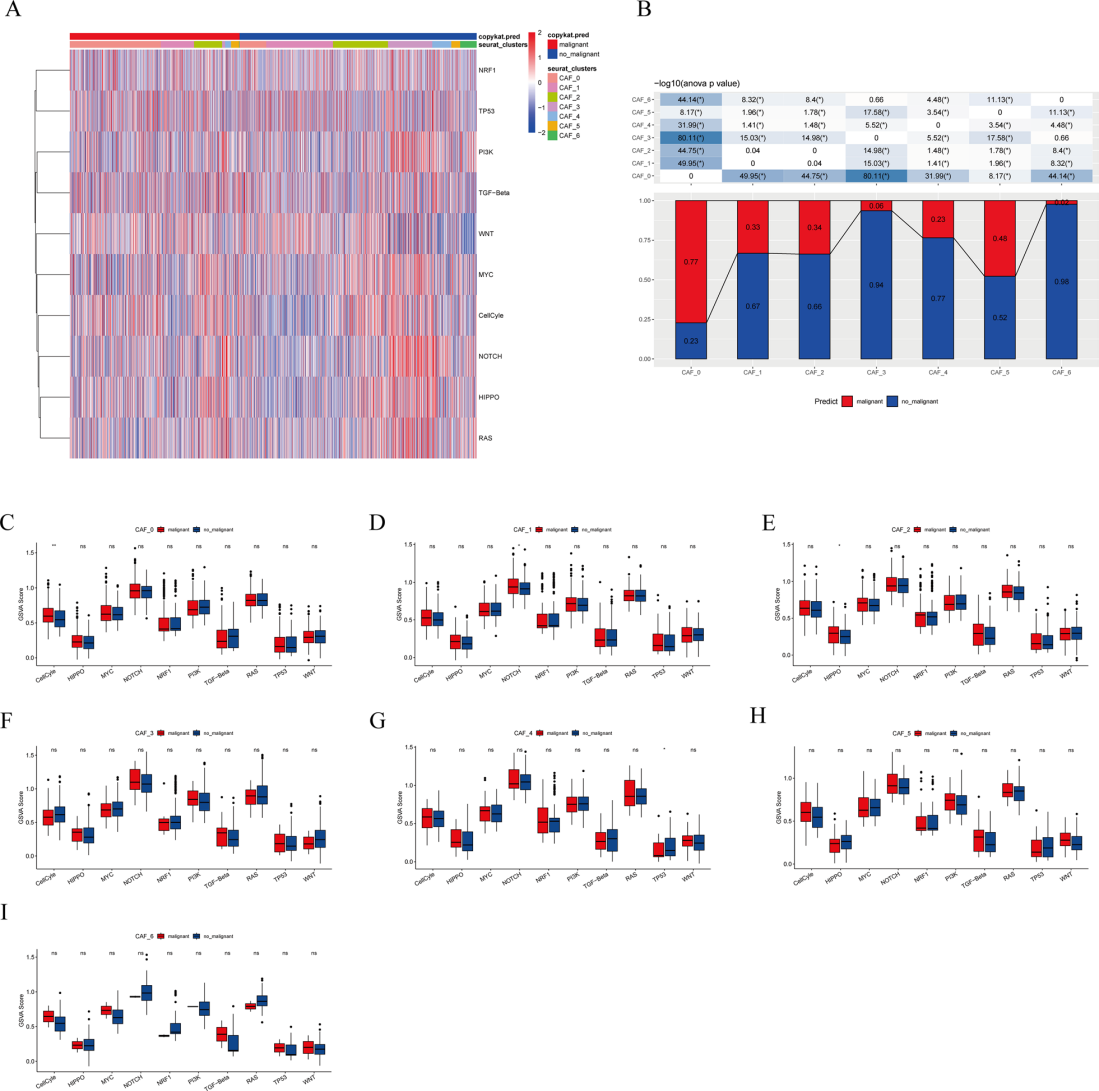
Attributes of cancer-associated pathways in clusters of CAF. **A** Heatmap displaying the scores of ten cancer-associated pathways in CAF cells; **B** comparative analysis between malignant cells and non-malignant cells in **C** CAF_0, **D** CAF_1, **E** CAF_2, **F** CAF_3, **G** CAF_4, **H** CAF_5, and **I** CAF_6 clusters based on the GSVA scores of each pathway. Wilcox test; *P < 0.05, **P < 0.01; ***P < 0.001; ****P < 0.0001; *ns* not significant

The association of CAF clusters with cancer progression was explored by analyzing the features of ten tumor-associated pathways found in the seven CAF clusters. Figure 3A shows the GSVA scores of these pathways in different CAF clusters. The CAF_0 cluster was observed to have a substantially higher ratio of malignant cells than the other six clusters (Fig. 3B). The CAF_5 cluster ranked second in the proportion of malignant cells, whereas the CAF_3 and CAF_6 clusters had the lowest proportion of malignant cells (Fig. 3B). Additionally, the GSVA scores of the ten tumor-linked pathways were compared between malignant and non-malignant cells found in every CAF cluster, as depicted in Fig. 3C–I.

**Fig. 4:**
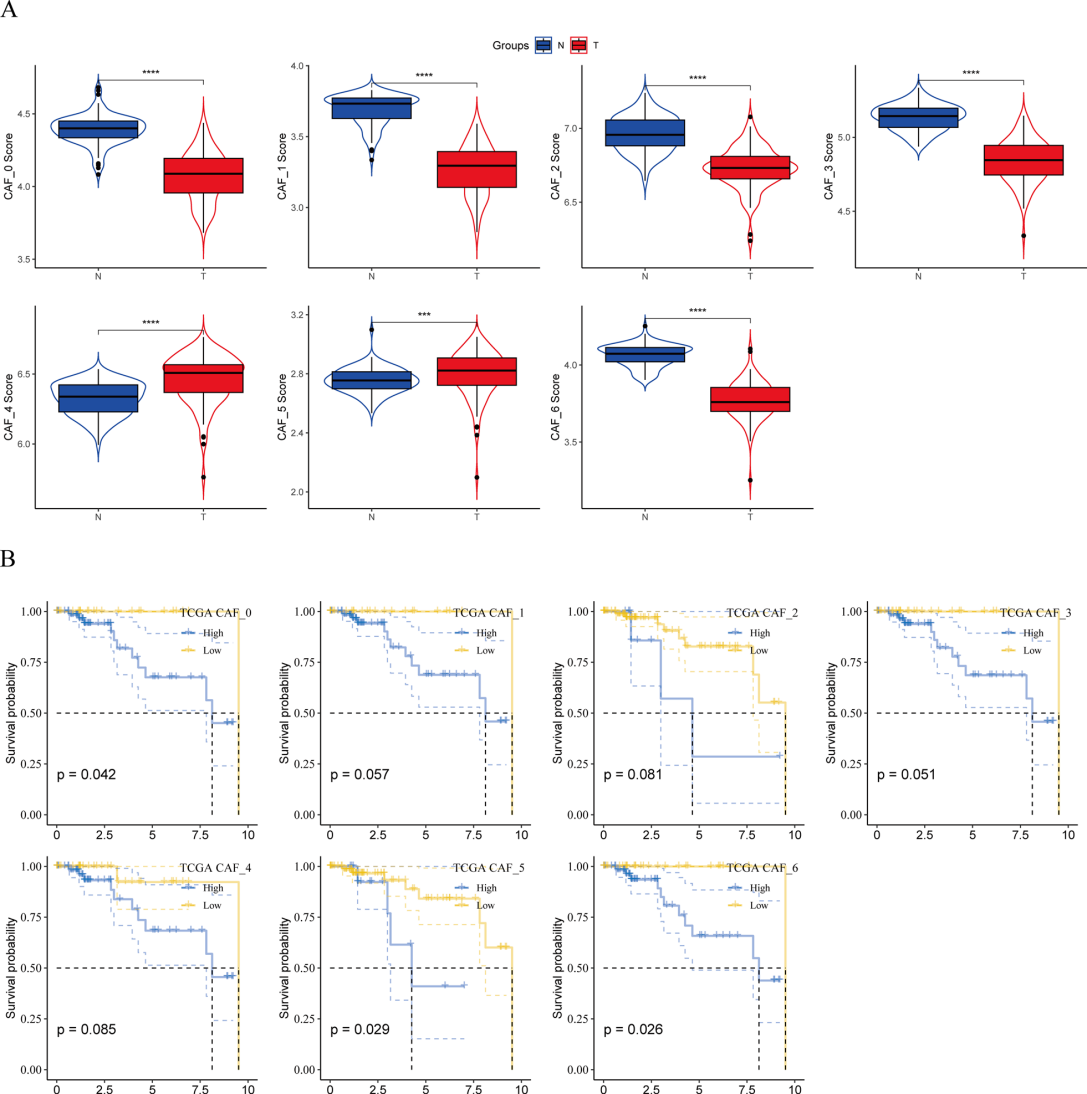
Links between the seven CAF clusters and the prognosis of patients with TNBC. **A** Comparative analysis of the seven CAF scores between cancerous and healthy tissues; **B** K–M curves analyzing the groups with high and low CAF scores in the seven clusters. ****P < 0.0001

To assess the association of CAF clusters with prognosis, the single-sample gene set enrichment analysis (ssGSEA) score of the marker genes (the top five DEGs of CAF clusters as outlined in Fig. 2C) for each CAF cluster was calculated employing the TCGA cohort. The findings revealed that the scores in cancer samples were significantly elevated in the CAF_5 and CAF_6 clusters in comparison to those in healthy samples. On the contrary, the remaining CAF clusters showed a reverse trend, with higher scores in healthy samples compared to tumor samples, as illustrated in Fig. 4A.

Using the optimal cut-off value determined with the survminer R package, high-CAF score and low-CAF score groups were identified from the TNBC samples in the TCGA dataset. An improved prognosis was observed in the high-CAF score group in comparison to that in the other group in the CAF_0, CAF_5, and CAF_6 clusters. However, no association was observed between the CAF_1, CAF_2, CAF_3, and CAF_4 clusters and the prognosis of patients with TNBC (Fig. 4B, E).

These findings suggest that while the enrichment of CAF clusters (1–4) differs between TNBC and normal samples, their contribution to TNBC progression may be limited.

### Detection of CAF-related hub genes

**Fig. 5:**
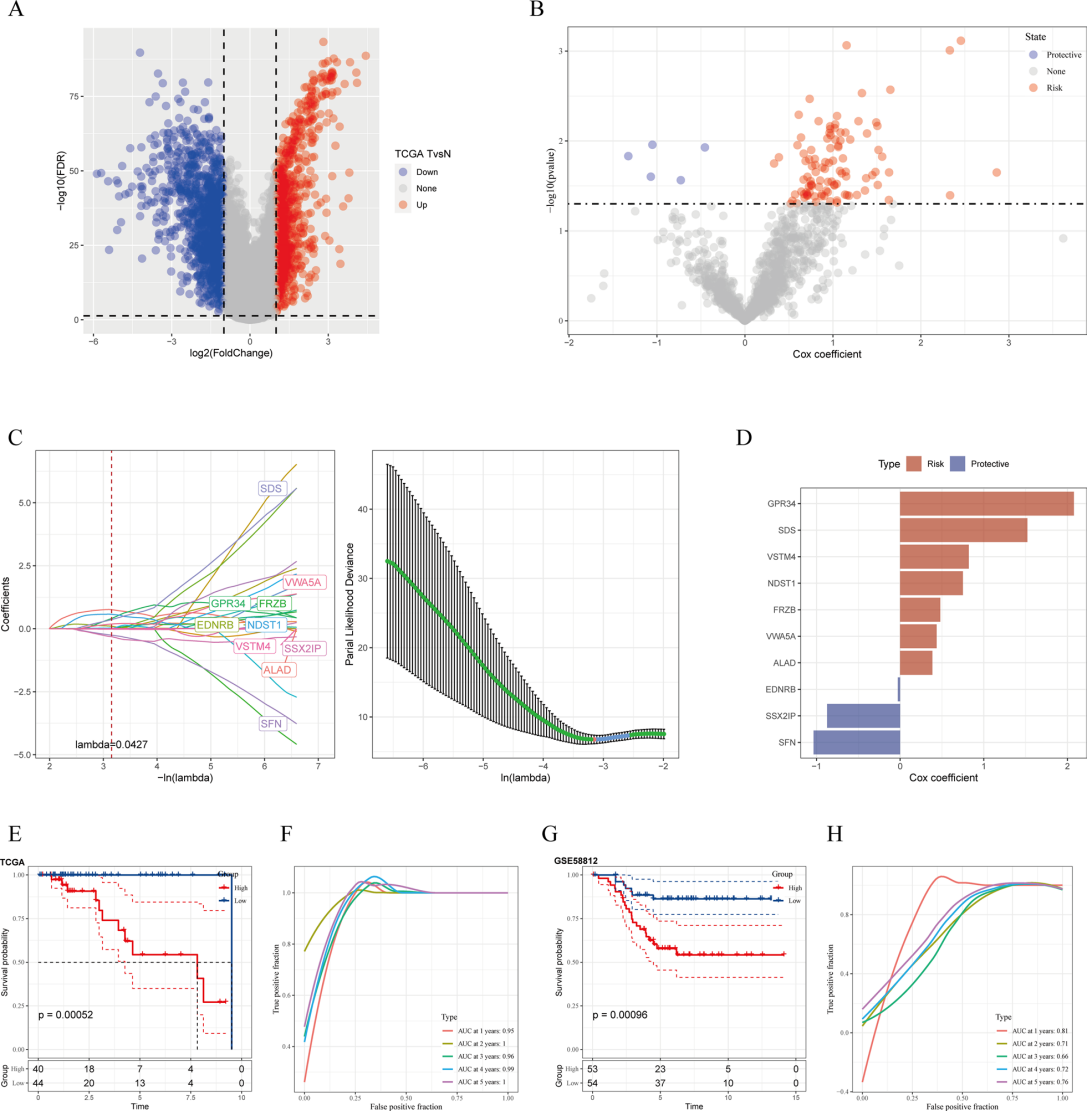
Detection of hub predictive genes for developing the risk signature. **A** Volcano plot of DEGs between cancerous and healthy tissues in TCGA cohort; **B** volcano plot of prognosis-linked genes determined using the univariate Cox regression analysis; **C** trajectory of each independent variable with lambda, and plots of the coefficient distributions generated for the logarithmic (lambda) series used for parameter selection (lambda); **D** multivariate Cox coefficients for every risk signature gene. **E**, **G** K–M curves of the developed risk model based on the ten genes in the TCGA and GEO cohorts. **F**, **H** ROC curves of the developed risk model based on the ten genes in the TCGA and GEO cohorts

A risk signature was constructed by first screening the DEGs between cancer and healthy tissues. A total of 2160 DEGs (FC > 1, *P* < 0.05) were identified, which included 893 upregulated and 1267 downregulated DEGs, as shown in Fig. 5A. Among them, 1180 genes exhibited a significant association with prognosis-linked CAF clusters. In addition, by evaluating the prognosis-predictive value of each gene employing univariate Cox regression analysis, 106 genes with significant prognosis-predictive value were detected (Fig. 5A, B). Subsequently, a subset of genes was identified through Lasso-Cox regression analysis, yielding ten genes with a lambda value of 0.0427 (Fig. 5C).

Finally, ten genes, including G protein-coupled receptor 34 (*GPR34*), serine dehydratase (*SDS*), V-set and transmembrane domain containing 4 (*VSTM4*), *N*-deacetylase and *N*-sulfotransferase 1 (*NDST1*), frizzled-related protein (*FRZB*), von Willebrand factor A domain containing 5 A (*VWA5A*), aminolevulinate dehydratase (*ALAD*), endothelin receptor type B (*EDNRB*), and SSX family member 2 interacting protein (*SSX2IP*), and stratifin (SFN) were included in the risk signature following a multivariate Cox regression analysis using the stepwise regression technique (Fig. 5D).

A final ten-genes signature formula was obtained: Risk Score = − 0.028 * *EDNRB* + 0.480 * *FRZB* + 2.077 * *GPR34* + 0.750 * *NDST1 − *0.874 * *SSX2IP* + 0.819 * *VSTM4* + 0.436 * *VWA5A* + 1.521 * *SDS* + 0.386 * *ALAD* − 1.036 * *SFN*. The risk score was calculated for every sample. After z-mean normalization, samples were classified into high- and low-risk groups. Survival analysis employing the Kaplan–Meier technique indicated a significant difference in survival outcomes between both risk groups within the GEO and TCGA cohorts, with the former demonstrating poorer outcomes (Fig. 5E, G). The area under the ROC curve (AUC) values of the model for 1- to 5-year survival ranged from 0.66 to 0.81 and 0.95 to 1 in the GEO and TCGA cohorts, respectively (Fig. 5F, H).

### Responsiveness of the risk signature to PD-L1 blockade immunotherapy

T-cell immunotherapy has emerged as an anticancer therapy, improving survival outcomes [20]. The predictive significance of the risk signature was assessed for immune checkpoint therapy utilizing the IMvigor210 and GSE78220 cohorts. The former cohort consisted of 348 diseased individuals who exhibited varying responses, including complete response (CR), partial response (PR), stable disease (SD), and progressive disease (PD), to anti-PD-L1 receptor blockers.

**Fig. 6:**
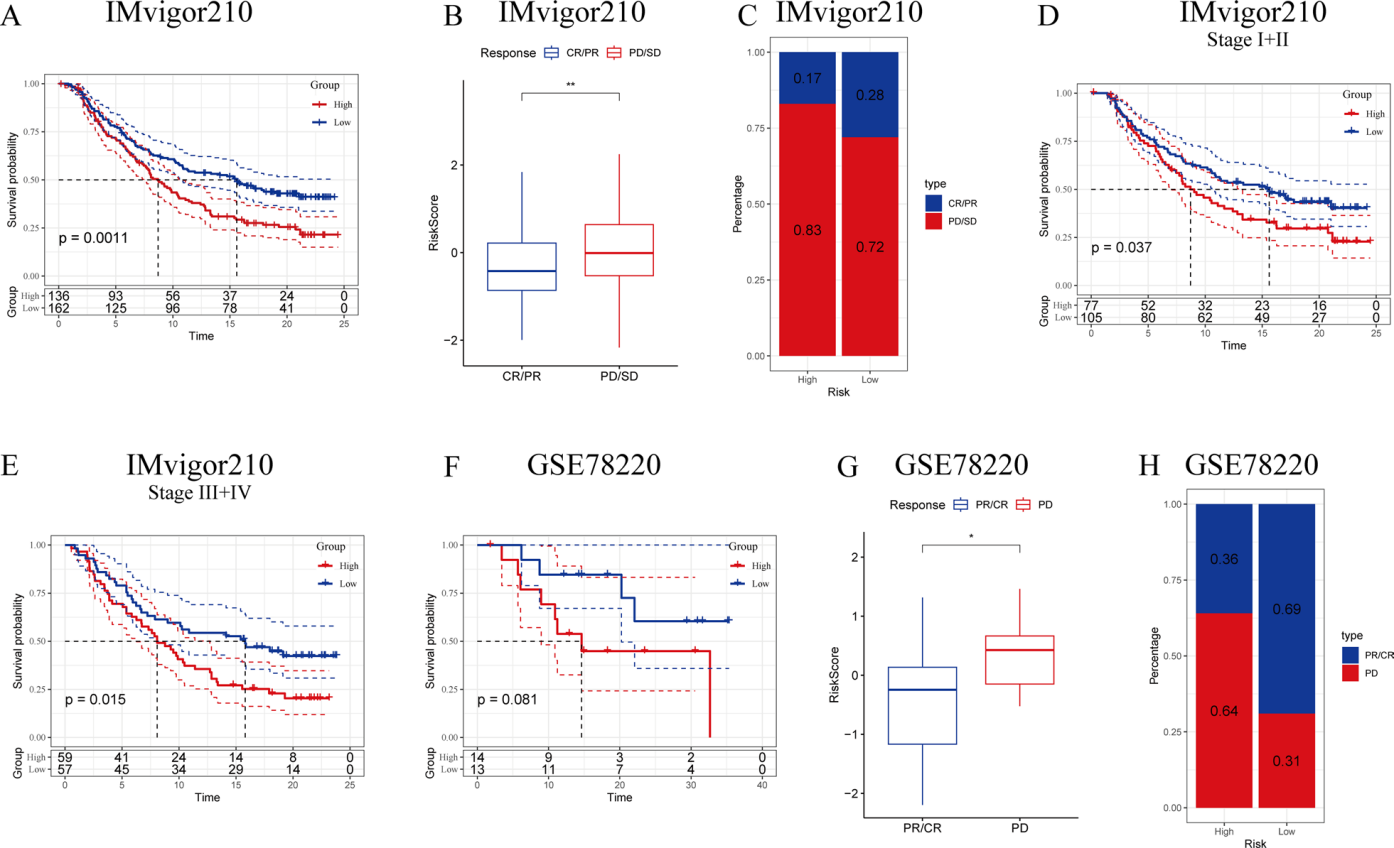
Responsiveness of risk score to PD-L1 blockade immunotherapy in the IMvigor210 cohort. **A** Prognostic differences between risk score groups in the IMvigor210 cohort. **B** Risk score differences in immunotherapy responses in the IMvigor210 cohort; **C** distribution of immunotherapy responses between risk score groups in the IMvigor210 cohort; **D** prognostic differences between risk score groups in early-stage patients in the IMvigor210 cohort; **E** prognostic differences between risk score groups in advanced-stage patients in the IMvigor210 cohort; **F** prognostic differences in risk score groups in the GSE78220 cohort; **G** risk score differences in immunotherapy responses in the GSE78220 cohort; **H** distribution of immunotherapy responses between risk score groups in the GSE78220 cohort. *P < 0.05; **P < 0.01

Substantial clinical advantage and significantly prolonged overall survival were recorded in low-risk patients of the IMvigor210 cohort compared to their high-risk counterparts (Fig. 6A, P = 0.0011). In addition, it was observed that SD/PD patients had risk scores exceeding those of CR/PR patients (Fig. 6B). The ratio of SD/PD patients was found to be increased in the high-risk group in comparison to the other group (Fig. 6C). significant differences in survival between the two risk groups were observed in Stage I + II (Fig. 6D, P = 0.037) and Stage III + IV (Fig. 6E, P = 0.015) patients, depicting a higher sensitivity of the risk score in patients at earlier stages. In the GSE78220 cohort, no significant difference in overall survival was observed between the two groups (Fig. 6F, P = 0.081). However, patients with PD exhibited higher risk scores in comparison to the CR/PR patients (Fig. 6G), with a greater proportion of PD patients in the high-risk group in comparison to that in the other group (Fig. 6H).

### Immune landscape and molecular expression profiles of the group

**Fig. 7:**
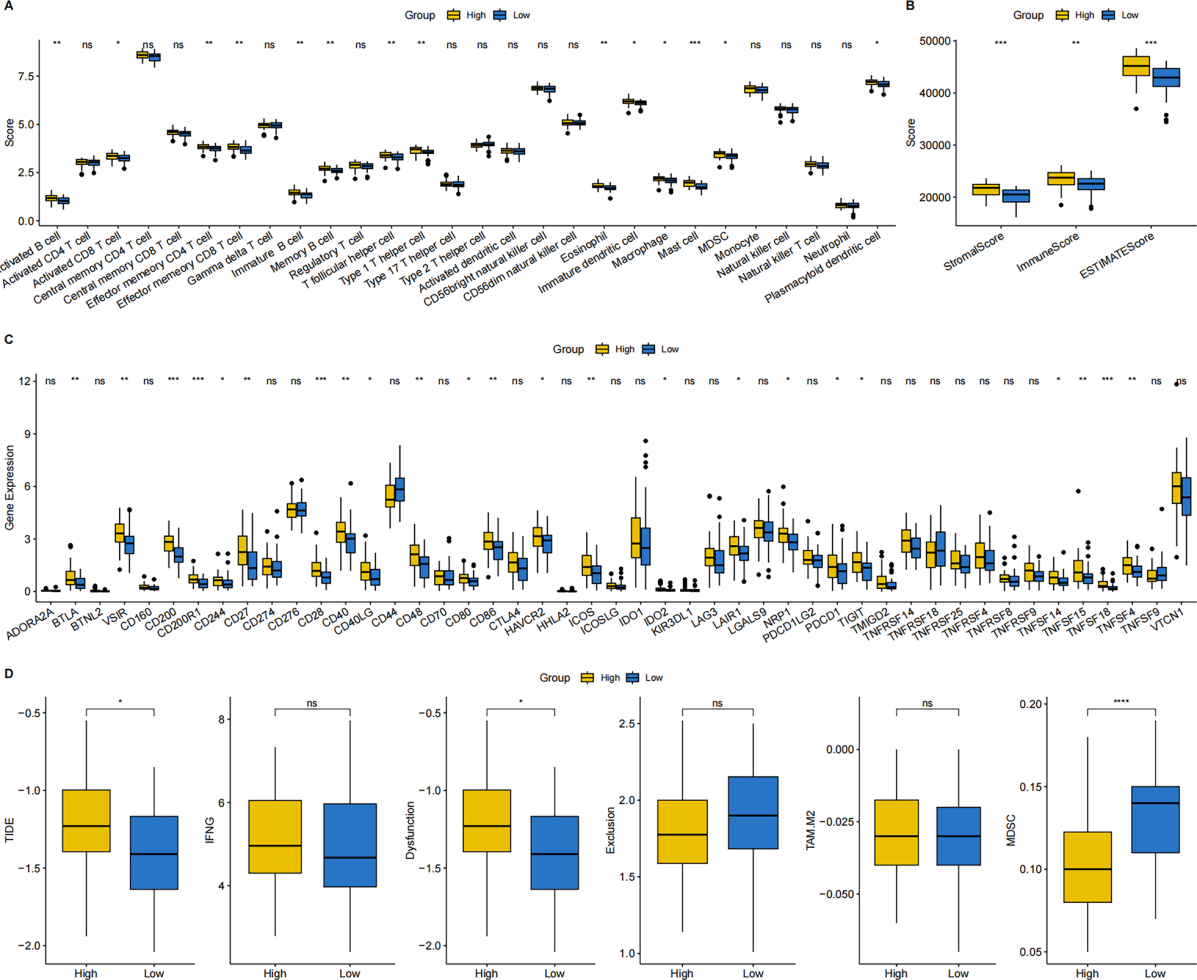
Immune landscape and molecular expression profiles of the two risk groups. **A** Differences in immune cell infiltration between the two risk groups; **B** stromal, immune, and ESTIMATE scores in the two risk groups; **C** expression of 47 ICMs in the two risk groups; **D** differences in metabolic and molecular subtypes in immunotherapy between the two risk groups

This study scrutinized the immune landscape and the expression of immune checkpoint molecules (ICMs) in both groups. Using ssGSEA, a higher infiltration abundance of various immune cell types, such as activated B and CD8 T, effector memory CD4 T and CD8 T, and mast cells, was detected in the high-risk group in comparison to that in the other group (Fig. 7A, B). Additionally, among the 47 included ICMs, the high-risk group showed increased relative expression levels of CD200, CD200R1, CD28, CD40, CD40LG, TNFSF14, TNFSF15, TNFSF18, and TNFSF4 (Fig. 7C). The results consistently indicate a higher likelihood of response to immunotherapy in high-risk patients with TNBC.

Differences between both groups were analyzed in terms of metabolic and molecular subtypes, Th1/IFN γ expression, predictive T cell dysfunction scores, and T cell rejection scores of metabolic and molecular subtypes TAM.M2 and MDSC within the context of immunotherapy. It was noted that the high-risk group presented significantly higher scores concerning metabolic and molecular subtypes in immunotherapy, as well as T-cell dysfunction in comparison to those in the other group. However, in the context of MDSC, the scores were lower in high-risk patients compared to those in low-risk patients (Fig. 7D).

**Fig. 8:**
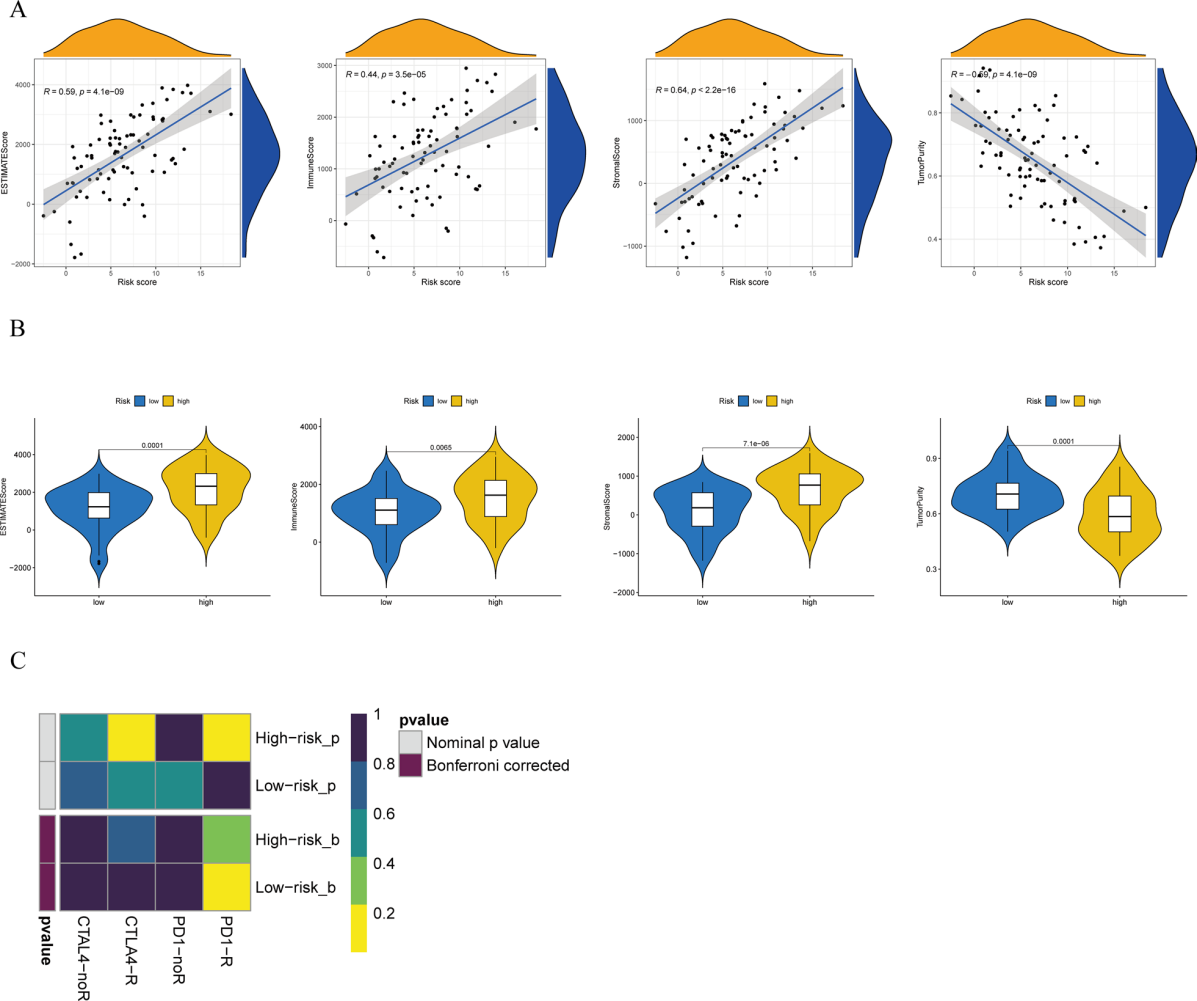
**A** Association of risk score with ESTIMATE, immune, and stromal scores and tumor purity. **B** Differences in ESTIMATE, immune, and stromal scores and tumor purity ratings between the two risk groups. **C** TIDE algorithm used to predict the probability of response to immune checkpoint inhibitor immunotherapy, categorizing patients into the two risk groups

Next, the association of risk stratification with the ESTIMATE, immune and stromal scores, and tumor purity were investigated (Fig. 8A). Risk stratification and the aforementioned indicators were significantly correlated. Further differences between both risk groups were assessed (Fig. 8B), revealing that the high-risk group had significantly increased ESTIMATE, immune, and stromal scores. In contrast, the low-risk group had significantly elevated tumor purity. The findings suggest a strong association of risk scores with immune status, exhibiting significant differences between both risk groups. The TIDE algorithm was utilized to estimate the potential response to immune checkpoint inhibitor immunotherapy in both risk groups (Fig. 8C).

**Fig. 9:**
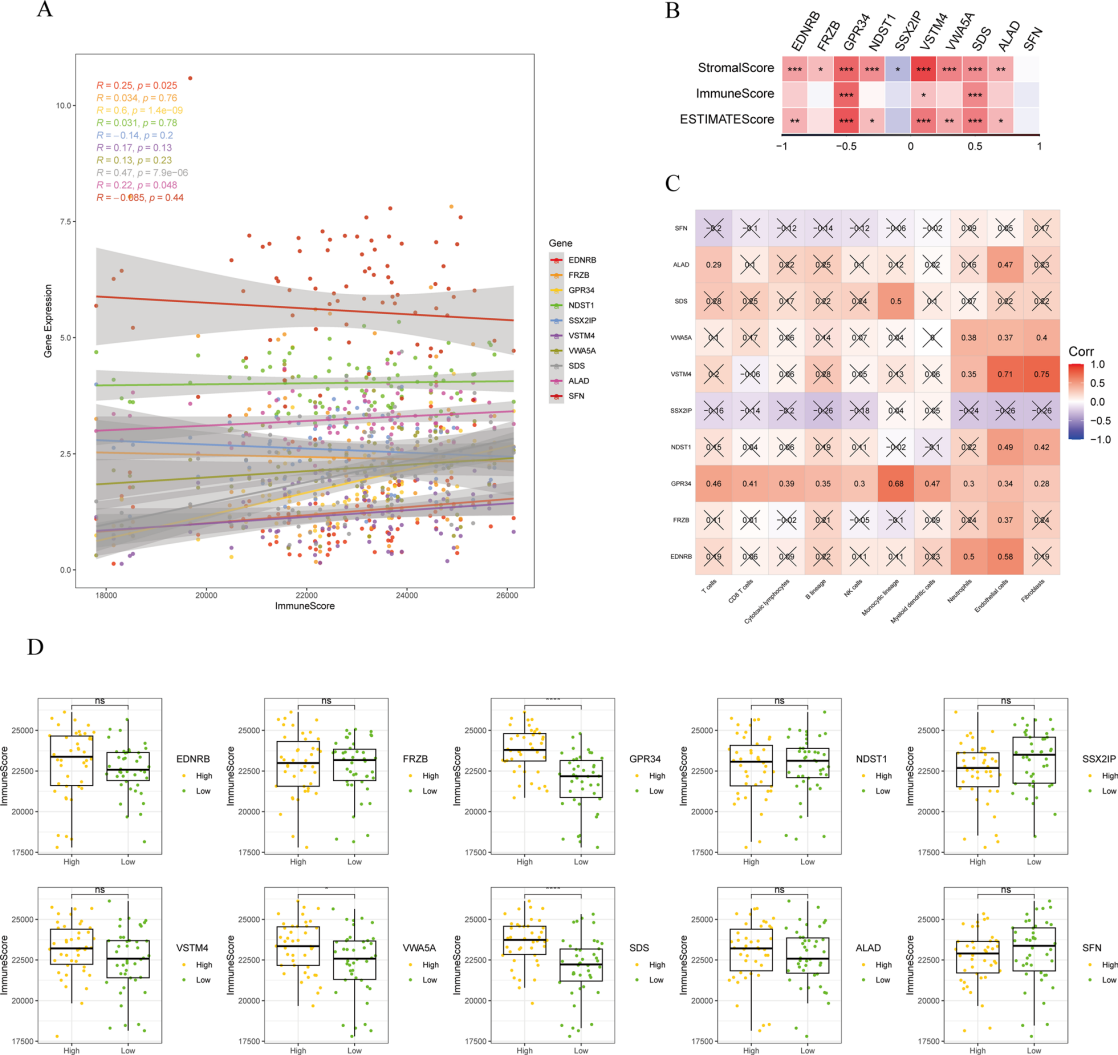
**A**, **B** Association of ten identified hub genes with immune infiltration. **C** Correlation of the ten hub genes with infiltration of various immune cells. **D** Differences in immune infiltration between different gene expression groups

The analysis focused on the possible pathways linked to the ten hub genes. In total, 21 pathways exhibited significant correlations with these hub genes, including the cell adhesion molecules, B cell receptor signaling pathway, FC gamma R-mediated phagocytosis, and leukocyte transendothelial migration (Additional file 2: Fig. S2). Moreover, the link between the determined hub genes and immune infiltration was examined. A substantial association was identified between *GPR34*, *SDS*, and immune infiltration (R > 0.3, *P* < 0.001; Fig. 9A, B). Additionally, a strong link was observed between GPR34 and various immune cells (Fig. 9C). Finally, the immune infiltration patterns in different gene expression groups were elucidated (Fig. 9D).

### Efficacy of risk grouping signature in drug sensitivity prediction

**Fig. 10:**
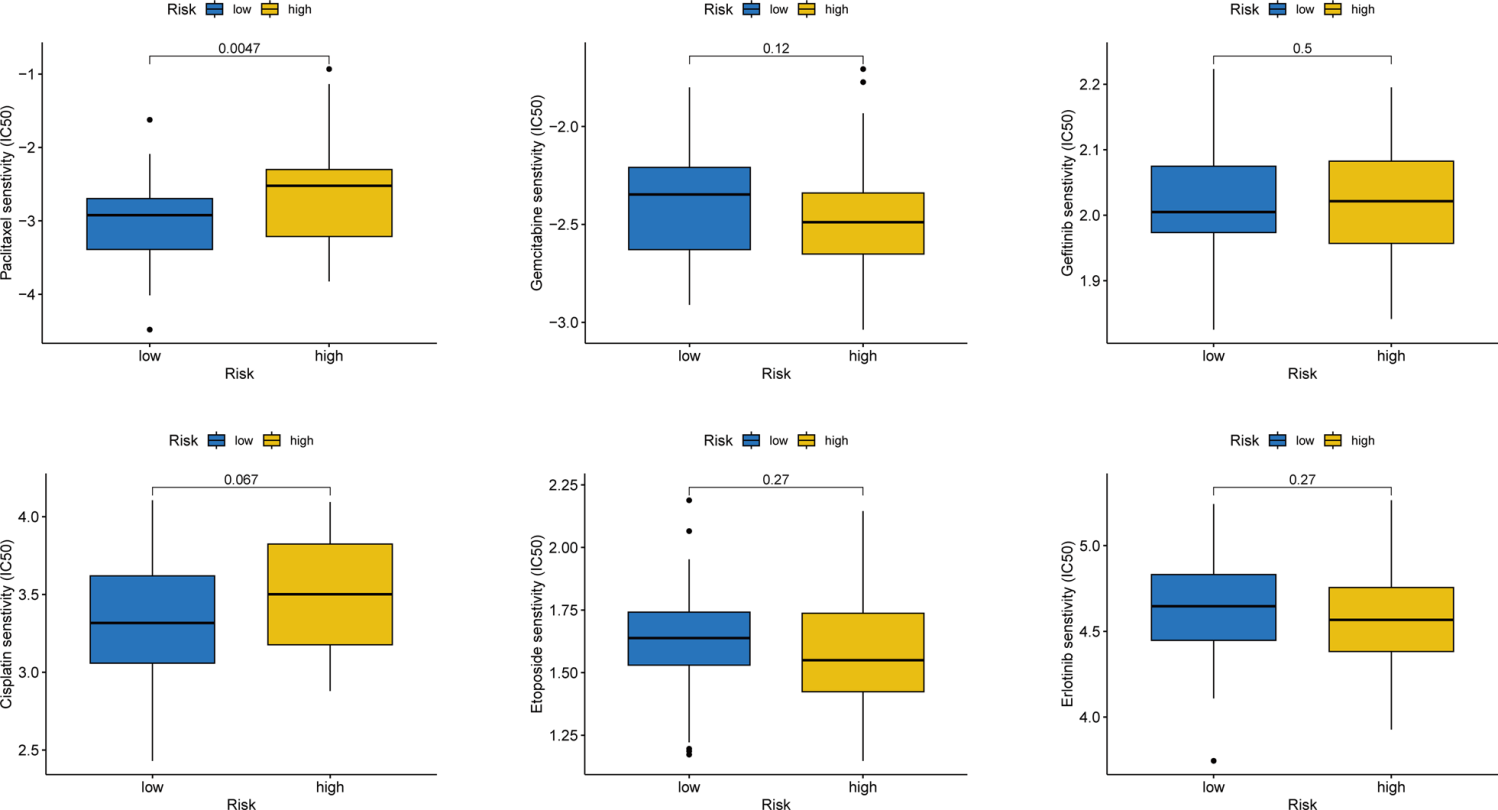
Efficacy of risk grouping signature in predicting drug sensitivity

To examine the association of the model with drug sensitivity, the half-maximal inhibitory concentration value for each drug was calculated in TNBC samples to identify any significant differences. Initially, differences in sensitivity to different drugs between the two groups were recorded, and it was noted that the high-risk group showed higher sensitivity to paclitaxel in comparison to the other group (Fig. 10). Additionally, this study explored the correlation between different hub genes and drugs, revealing strong associations between the enriched hub genes (such as *EDNRB* and *SFN*) and different drugs (Additional file 3: Fig. S3).

### GPR34 overexpression in TNBC tissue

**Fig. 11:**
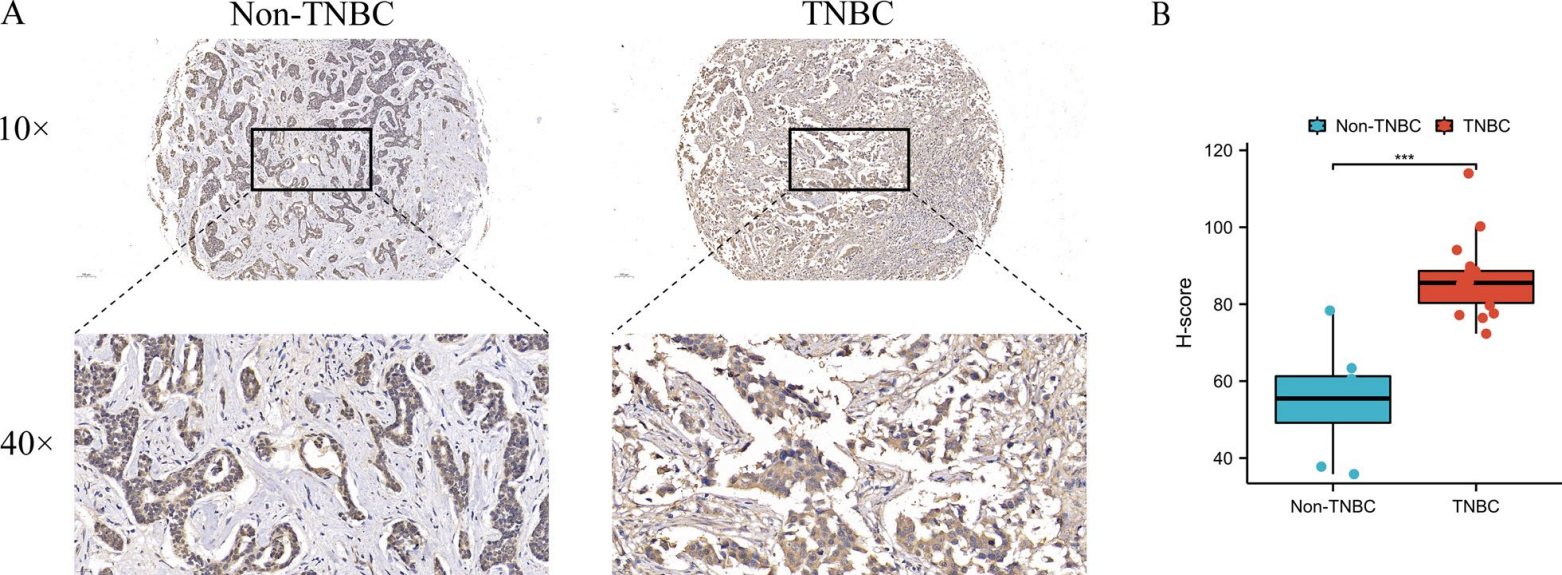
**A** Immunohistochemical expression of GPR34 in TNBC tissue; **B** GPR34 expression level in TNBC tissue was substantially elevated in comparison to tissue of non-TNBC patients

For scrutinizing the function of hub genes in breast cancer cells, tissue samples from 18 TNBC patients and 8 non-TNBC patients were collected. Additionally, IHC was conducted to assess GPR34 expression in the tissue samples. The results indicated a significant increase in GPR34 expression in TNBC tissue samples in comparison to that in the tissue samples from non-TNBC patients (Fig. 11A, B).

## Discussion

Globally, breast cancer stands as the most common cancer among women, with nearly 2.3 million new cases diagnosed annually, making up 11.7% of all cancer cases [21]. The most aggressive subtype is TNBC, comprising 15–25% of all breast cancer cases. This type presents with the absence of ER, PR, and HER-2 expression, leading to poor response to endocrine or HER2-targeted therapy [22]. Therefore, identifying therapeutic targets and research strategies for TNBC is essential. There is a strong tendency for TNBC to metastasize, and patients who do not respond positively to chemotherapy typically experience a worse prognosis [23]. Current research on immune infiltration can predict the response of patients with TNBC to neoadjuvant chemotherapy and improve survival rates [24]. Immunotherapy, specifically immune checkpoint inhibitors, is a promising strategy for treating cancer [25, 26]. These inhibitors can suppress tumor growth by regulating the TME and immune cell function. However, it can benefit only a small number of patients with TNBC [27, 28], necessitating further research to enhance their response to immunotherapy.

CAFs, key components of the TME, have great research potential in tumor onset and progression. They are associated with various types of cancers, such as prostate [29], ovarian [30], pancreatic [31], and gastric [32] cancers. Furthermore, TNBC exhibits a strong correlation with CAFs. In TNBC, CAFs can induce the formation of lipid-associated macrophages and mediate immune suppression [33]. Furthermore, CAFs are involved in promoting immune escape in TNBC [34]. In the present research, seven subtypes of CAFs were identified through CAF subtyping analysis, and three of them were associated with the prognosis of patients with TNBC. Through differential gene enrichment analysis of CAFs and TNBC, a characteristic risk model of CAFs, comprising ten relevant genes was established. The model demonstrated good predictive ability, with a predictive efficacy greater than 0.9 in the TCGA database and a validation efficacy greater than 0.8 in the external dataset GSE58812.

Previous research has seen scholars employ comprehensive analysis of various cell death modes to establish a novel predictive model, capable of accurately predicting the clinical prognosis and drug sensitivity of TNBC post-surgery [35]. Notably, the focus on necrotic apoptosis has attracted the attention of many scholars, leading to the development of relevant predictive models and classifications based on necrotic apoptosis-related genes, which have also demonstrated good efficacy in predicting prognosis [36]. Similar predictive models for TNBC have been extensively researched, such as Exosome-Related Gene [37], m5C RNA Methylation Regulators [38], T cell-related [39], and Homologous Recombination Deficiency [40], among which the Homologous Recombination Deficiency score has been confirmed to be effective in predicting the response of triple-negative breast cancer patients to platinum-based neoadjuvant chemotherapy.

Previous research has also grouped BC by examining the gene expression profiles of CAF cells, revealing that the gene profiles of each CAF subtype are associated with unique functional programs and exhibit excellent prognostic capabilities in clinical cohorts [41]. Additionally, other research has used single-cell analysis and machine learning to identify risk prognostic features based on cancer-associated fibroblast characteristic genes, which can guide immunotherapy for BC patients [42, 43]. In summary, risk assessment models established through the analysis of CAF cells have shown significant potential for the treatment of BC patients. To our knowledge, we are the first to construct a predictive model for TNBC using single-cell bioinformatics technology in combination with CAF-related genes. Importantly, this predictive model has demonstrated excellent efficacy in the validation set.

Using the risk model established by CAFs, the efficacy of immune checkpoint therapy in the IMvigor210 cohort was validated. Patients in the low-risk group patients exhibited significant clinical benefits and longer overall survival compared to those in the high-risk group. Furthermore, significant differences in the immune landscape and ICM expression were observed between both groups, with the high-risk group exhibiting a relatively higher abundance of immune cell infiltration. These findings suggest that CAF-based classification can potentially improve the effectiveness of immunotherapy.

The potential pathways associated with the ten hub genes were analyzed, and 21 significantly associated pathways were identified. They included several immune response-associated pathways, including the FC gamma R-mediated phagocytosis, leukocyte transendothelial migration, and the B cell receptor signaling pathway. Moreover, several genes were found to be associated with [44–50], with GPR34 showing carcinogenesis, with GPR34 exhibiting a close association with various types of cancer, such as gastric and cervical cancers.

*GPR34* was validated as the gene with the strongest association with TNBC prognosis. A significantly high expression level of GPR34 was observed in TNBC tissues. Reportedly, knocking down GPR34 expression can inhibit the proliferation and migration of gastric cancer cells [51], and GPR34 regulates metabolism and metastasis in gastric cancer [44]. Consistent with the findings of this research, GPR34 has been involved in the immune response in various tumors [52–54]. A strong correlation was observed between GPR34 and immune infiltration, with high GPR34 expression in patients harboring high immune infiltration levels, particularly in the monocytic lineage. GPR34 functions as a G protein-coupled receptor that can activate classic signaling pathways of related G protein families, including inhibiting adenylyl cyclase and activating phospholipase C-IP3/diacylglycerol, PI3K-AKT, and RAS-ERK pathways [55]. Furthermore, GPR34 influences the responsiveness of the immune system to pathogens [56].

When it comes to the other model genes, we intend to conduct experimental validation analyses in subsequent experiments. Furthermore, through literature research, we have discovered that many of the genes in our model are closely linked to cancer development. For instance, NDST1 [57], FRZB [58], ALAD [59], EDNRB [60], SSX2IP [61], and SFN [62] have strong associations with breast cancer development, which bolsters our confidence in the viability of our model. Regarding the remaining model genes, like VSTM4 [46] and VWA5A [63], they have also been reported to have connections with cancer. However, their precise roles in cancer development, especially their interactions with genes like SDS, remain unclear. This information highlights the potential significance of these genes in cancer, and we are eager to delve deeper into their roles in our future research.

This study has certain limitations. We performed a dataset analysis in the database using bioinformatics techniques. Hence, in future research, it is imperative to delve deeper into the biological functions of CAF cells and their associated genes. Furthermore, the number of TNBC samples used for validation was relatively small, necessitating an expansion of the sample size in subsequent studies.

## Conclusion

This study systematically generated and evaluated a risk score for TNBC based on ten CAF-related genes. This model was correlated with the TME and could predict the prognosis of patients and their responses to immunotherapy. Additionally, the hub genes were validated in TNBC tissues. Taken together, the findings of this study provide novel research ideas and therapeutic targets for TNBC.

### Supplementary Information

**Additional file 1: Figure S1.** Clustering and dimensionality reduction of CAF populations. A: Distribution of subgroups following cell clustering. B: t-SNE map of the expression pattern of fibroblast marker genes. C: Distribution of subgroups following fibroblast re-clustering. D: t-SNE maps of marker gene expression in seven CAF clusters.

**Additional file 2: Figure S2.** A, B: Correlation of ten hub genes with different pathways; C, D: GSEA enrichment showing the most differentiated correlated pathways in the two risk groups.

**Additional file 3: Figure S3.** A: Analysis of the association between various genes and drug sensitivity. B: Group exhibiting a gene-drug correlation greater than 0.4.

**Additional file 4: Table S1.** Datasets related details of GSE199515, GSE58812, GSE78220 and IMvigor210.

## Data Availability

The datasets used and analyzed in this study are available from the GEO dataset (https://www.ncbi.nlm.nih.gov/geo/). The data that support the findings of this study are available on request from the corresponding author.
