# Supplementary figures and images for: Characterization of cancer-associated fibroblasts (CAFs) and development of a CAF-based risk model for triple-negative breast cancer
Source: Cancer Cell Int. 2023 Nov 25;23:294. doi: 10.1186/s12935-023-03152-w (PMC10676599; doi:10.1186/s12935-023-03152-w)

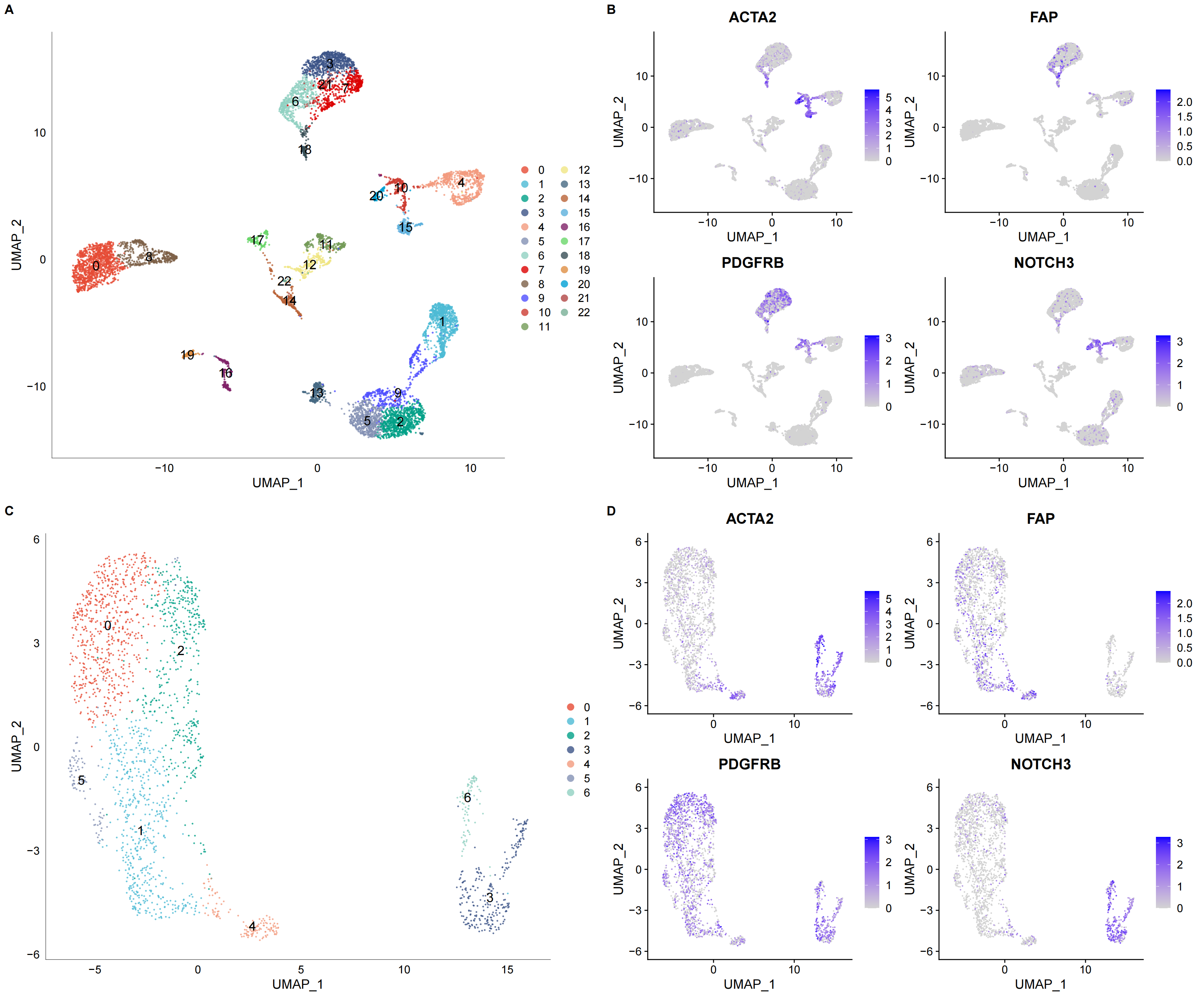

Supplement: Supplementary file 1 — Additional file 1: Figure S1. Clustering and dimensionality reduction of CAF populations. A: Distribution of subgroups following cell clustering. B: t-SNE map of the expression pattern of fibroblast marker genes. C: Distribution of subgroups following fibroblast re-clustering. D: t-SNE maps of marker gene expression in seven CAF clusters. [file 12935_2023_3152_MOESM1_ESM.tif]

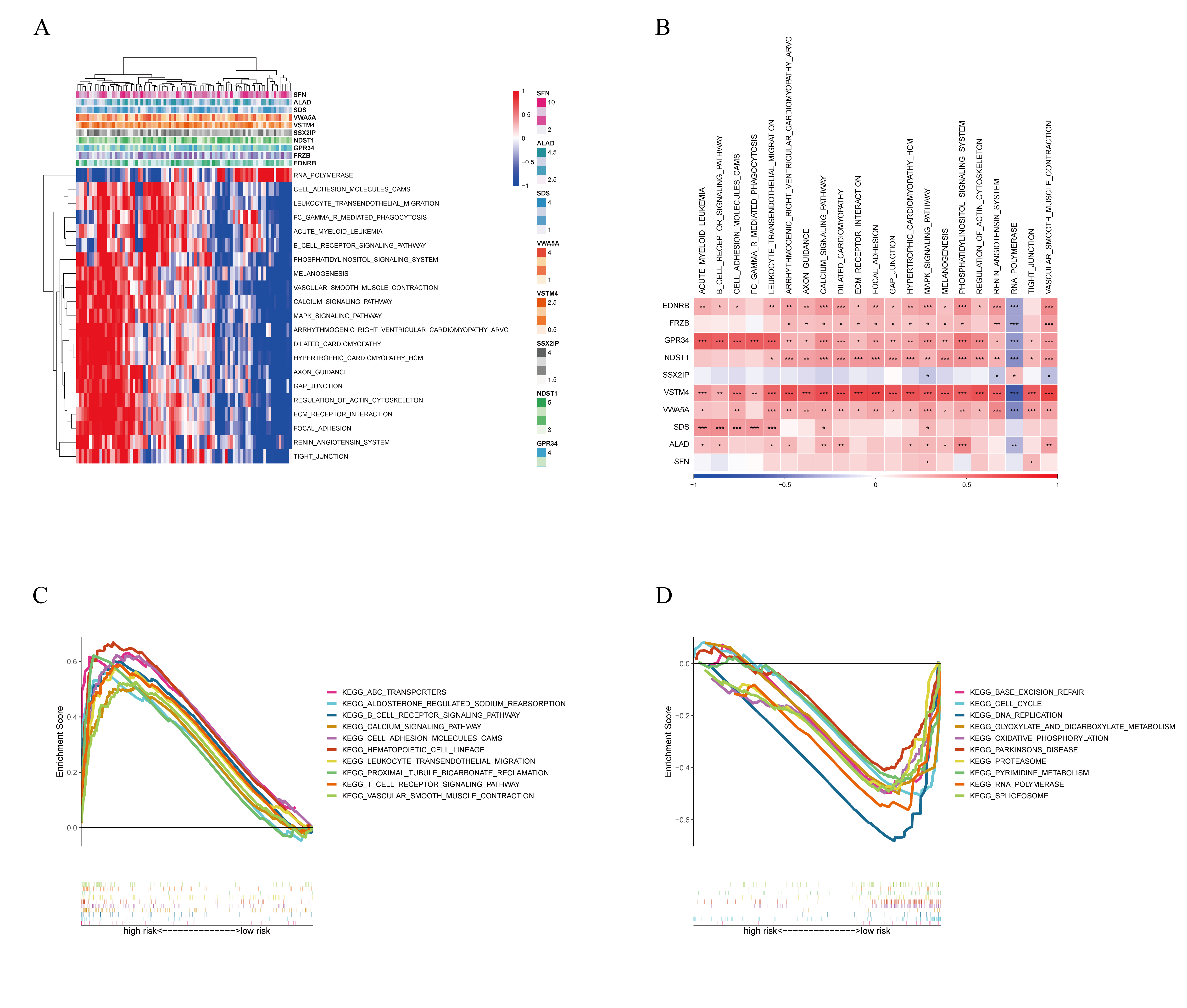

Supplement: Supplementary file 2 — Additional file 2: Figure S2. A, B: Correlation of ten hub genes with different pathways; C, D: GSEA enrichment showing the most differentiated correlated pathways in the two risk groups. [file 12935_2023_3152_MOESM2_ESM.tif]

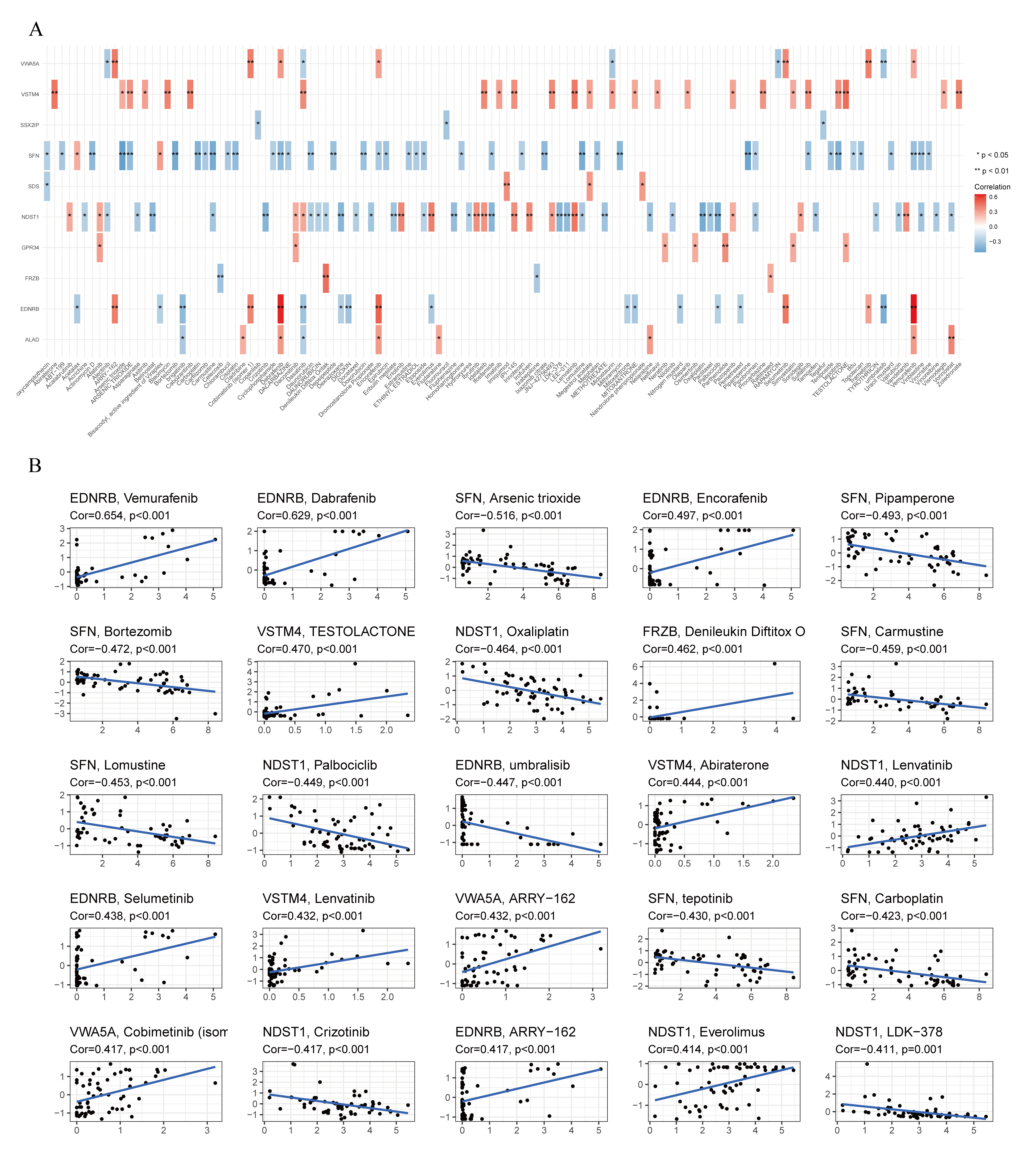

Supplement: Supplementary file 3 — Additional file 3: Figure S3. A: Analysis of the association between various genes and drug sensitivity. B: Group exhibiting a gene-drug correlation greater than 0.4. [file 12935_2023_3152_MOESM3_ESM.tif]
