# Supplementary material for: Characterization of cancer-associated fibroblasts (CAFs) and development of a CAF-based risk model for triple-negative breast cancer
Source: Cancer Cell Int. 2023 Nov 25;23:294. doi: 10.1186/s12935-023-03152-w (PMC10676599; doi:10.1186/s12935-023-03152-w)
Supplement: Supplementary file 4 — Additional file 4: Table S1. Datasets related details of GSE199515, GSE58812, GSE78220 and IMvigor210. [file 12935_2023_3152_MOESM4_ESM.docx]

Table S1

| **ID** | **Organisms** | **Samples Disease** | **Datatype** |
| --- | --- | --- | --- |
| GSE199515 | Homo sapiens; Mus musculus | 13(TNBC: three human samples and ten mouse samples) | Sc-RNA seq |
| GSE58812 | Homo sapiens | 107 (TNBC patients) | Expression profiling by array(IHC) |
| GSE78220 | Homo sapiens | 38 (Genomic and Transcriptomic Features of Response to Anti-PD-1 Therapy in Metastatic Melanoma) | Expression profiling by high throughput sequencing(Training) |
| IMvigor210 | Homo sapiens | the IMvigor210 dataset contains the microarray, survival and anti-PD-L1 immunotherapy data of metastatic urothelial cancer patients | The transcriptome data and clinical data (Training) |
